# Chromosome-level genome assembly of the caenogastropod snail *Rapana venosa*

**DOI:** 10.1038/s41597-023-02459-7

**Published:** 2023-08-16

**Authors:** Hao Song, Zhuoqing Li, Meijie Yang, Pu Shi, Zhenglin Yu, Zhi Hu, Cong Zhou, Pengpeng Hu, Tao Zhang

**Affiliations:** 1 CAS Key Laboratory of Marine Ecology and Environmental Sciences, Institute of Oceanology, Chinese Academy of Sciences, Qingdao, 266071 China; 2 Laboratory for Marine Ecology and Environmental Science, Qingdao National Laboratory for Marine Science and Technology, Qingdao, 266237 China; 3 University of Chinese Academy of Sciences, Beijing, 100049 China; 4 Research and Development Center for Efficient Utilization of Coastal Bioresources, Yantai Institute of Coastal Zone Research, Chinese Academy of Sciences, Yantai, 264003 China

**Keywords:** Genome, Genomics

## Abstract

The carnivorous gastropod *Rapana venosa* (Valenciennes, 1846) is one of the most notorious ecological invaders worldwide. Here, we present the first high-quality chromosome-scale reference *R. venosa* genome obtained via PacBio sequencing, Illumina paired-end sequencing, and high-throughput chromosome conformation capture scaffolding. The assembled genome has a size of 2.30 Gb, with a scaffold N50 length of 64.63 Mb, and is anchored to 35 chromosomes. It contains 29,649 protein-coding genes, 77.22% of which were functionally annotated. Given its high heterozygosity (1.41%) and large proportion of repeat sequences (57.72%), it is one of the most complex genome assemblies. This chromosome-level genome assembly of *R. venosa* is an important resource for understanding molluscan evolutionary adaption and provides a genetic basis for its biological invasion control.

## Background & Summary

Caenogastropoda is an extraordinarily large and diverse group containing thousands of described species and comprising ~60% of extant gastropod species^1^. These snails are extremely diverse in morphology, diet, and habitat and inhabit marine, terrestrial, and freshwater environments in the wild^2,3^. To date, only two chromosome-level genomes of this clade have been published^4,5^, which limits our understanding of the internal phylogeny and evolutionary adaption of this important clade.

*Rapana venosa* (Valenciennes, 1846) is a common marine carnivorous snail in the Caenogastropoda. It is native to the coasts of the Bohai, East, and Yellow Seas in China, the northern Korean peninsula, the far east of Russia, and northern Japan^6^, and is an economically important species in China^7^. Via global transport, *R. venosa* has unintentionally been introduced into the Rio de la Plata between Argentina and Uruguay, Chesapeake Bay, Quiberon Bay in France, and the coastal waters of the Netherlands, as an invasive species^8–11^. Its successful establishment in these areas is based on its strong ecological fitness, involving high fecundity, easy dispersal as planktonic larvae, rapid growth rate, early sexual maturity, and broad tolerance to oxygen depletion, salinity, temperature, and water pollution^12^. In the Chesapeake Bay region, *R. venosa* has very different prey and predation strategies from the native gastropod, *Urosalpinx cinerea*, and therefore disrupts the local trophic structure and attenuation of native shellfish resources^13^. As *R. venosa* feeds on economically valuable bivalves, such as oysters, mussels, and clams, it has also caused severe economic losses in the Black Sea area^14^. The economic importance in Asian countries and global ecological invasiveness of this species has led to extensive studies on its developmental mechanism and the genetic basis of its environmental adaptation^15–17^. However, such studies are hampered by the lack of related genomic resources.

**Fig. 1:**
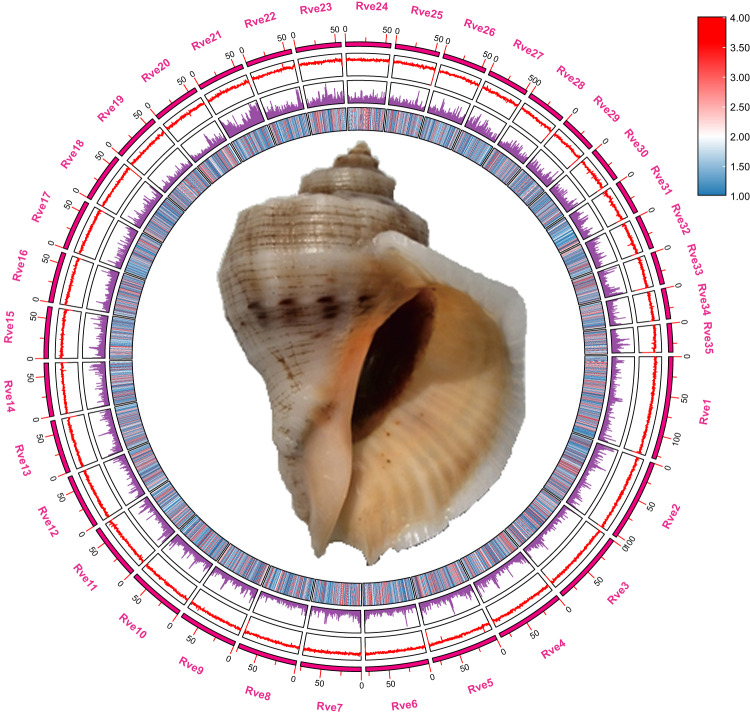
Characterization of assembled *R. venosa* genome. From inner to outer layers: photograph of *R. venosa*, gene abundance, repeat element abundance, GC rate, and chromosome-level scaffolds at scale.

In this study, we used short reads generated by an Illumina platform, long reads generated by PacBio sequencing, and high-throughput chromosomal conformation capture (Hi-C) analysis to construct a high-quality *R. venosa* reference genome at the chromosomal level (Fig. 1). The genome sequences were assembled into 17,949 contigs, with a contig N50 length of 434.10 kb and a total length of 2.30 Gb. Chromosome scaffolding resulted in 5,242 sequences corresponding to 35 chromosomes. The largest 35 chromosome scale scaffolds are in total 2.25 Gb long, which corresponds to 97.88% of the total contig length. Using *de novo* and homolog-based strategies, 29,649 protein-coding genes were revealed by gene annotation, 77.22% of which were annotated in the publicly available NCBI RefSeq non-redundant protein, KEGG, TrEMBL, Swissprot, and InterPro databases. The *R. venosa* genome assembly has a high heterozygosity of 1.41% and a large proportion of repeat sequences (57.72%) and, therefore, is one of the most complex genome assemblies. Phylogenetic analysis indicated that *R. venosa* speciated from the common ancestor of *Conus consors* approximately 124.4 mya (78.3–177.5 mya).

## Methods

### Sample collection and sequencing

Living specimens of *R. venosa* were collected from Laizhou Bay, China. We extracted genomic DNA from *R. venosa* muscle samples using a QIAGEN DNeasy Kit (QIAGEN, Shanghai, China) as per the product manual. We used electrophoresis on a 1% agarose gel to examine the quality of the isolated genomic DNA. To ensure the DNA samples met the sequencing requirements, we used a Qubit instrument to quantify the concentration and 23.2 ng/µL DNA was obtained. Then, the genomic DNA was purified and concentrated by AMpure PB magnetic beads. The processed genomic DNA were further applied to prepare a single-molecule real-time bell sequencing library using the SMRTbell Template Prep Kit 2.0 (Pacific Biosciences, Menlo Park, CA, USA)^18^. The library was sequenced using the Pacific Biosciences Sequel II in continuous long-read (CLR) mode following the manufacturer’s instructions. As a result, 3 SMRT cells were sequenced, and we obtained a total of 256.49 Gb PacBio reads. The N50 and N90 lengths of the reads were 434.10 kb and 58.92 kb, respectively. Based on the protocol, we constructed the Illumina short-insert (350 bp) library. Paired-end sequencing was performed on the Illumina Novaseq 6000 platform (Illumina, Inc., San Diego, CA, USA) and a total of 153.00 Gb reads were obtained. For the Hi-C sequencing, fresh muscle was fixed in 1% formaldehyde and the fixation was terminated with 0.2 M glycine. In accordance with the protocol^19^, we prepared the Hi-C library and then sequenced on an Illumina NovaSeq 6000 sequencing platform^19^.

### Genome assembly

*R. venosa* genome assembly was challenging because of the extremely high percentages of sequence repeats (57.72%) and heterozygosity (1.41%). We tried different genome-assembly strategies and ultimately selected that with the highest continuity and accuracy (Table 1). In total, 256.49 Gb of PacBio long-read data was used for *de novo* genome assembly using wtdbg v 2.4^20^, which resulted in 17,949 contigs and a contig N50 length of 434.10 Kb. We then used Pilon v 1.23^21^ to polish the assembled genome with the Illumina short reads from the same individual. Purge Haplotigs software was used to remove redundancy from the assembled genome, obtaining a 2,293.82 Mb long assembly (Table 2). The total gene space was 38.3 Mb and the mean exon number per mRNA was about six. In our previous genome survey analysis, the estimated genome size of *R. venosa* was 2.20 Gb with 67.04% sequence repeats using a *k*-mer analysis, quite near to the assembly in this study^22^. The genome assembly size of *R. venosa* is substantially larger than those of some closely related mollusc species, such as *Crassostrea gigas* (557.74 Mb)^23^, *Biomphalaria glabrata* (916.38 Mb)^24^, *Pomacea canaliculata* (440.07 Mb)^25^, and *Achatina immaculata* (1.65 Gb)^26^, similar to those of *Octopus bimaculoides* (2.40 Gb)^27^ and *Conus consors* (2.05 Gb)^5^, and smaller than that of *Conus bullatus* (3.43 Gb)^4^. Benchmarking Universal Single-Copy Orthologs (BUSCO) v 5.4.6^28^ was used to evaluate the completeness and quality of the *R. venosa* genome assembly against the metazoa_odb10 database. Of the 978 BUSCO orthologous groups, 886 (90.6%) were identified as complete in the assembled genome (Table 3). This assembly was even better than the recently published genome of another Neogastropoda member, *C. bullatus*, with a contig N50 length of 171.48 kb and a BUSCO (v 5.4.6) value of 89.8%^4^. The GC content of the *R. Rapana* genome assembly is 42.38%.

**Table 1:** Comparison of effects of different genome assembly schemes.

Method	Contig length	Contig N50	BUSCO
wtdbg2.4 + pilon + purge_haplotigs_full	2,295,076,713	434,100	90.60%
wtdbg2.0 + purge_haplotigs	3,815,562,603	944,770	83.20%
wtdbg2.4 + pilon + pilon	2,293,399,401	434.579	89.80%
wtdbg2.4 + pilon	3,105,793,653	239,999	90.10%
wtdbg2.0 + pilon	3,135,630,390	223,757	88.60%
wtdbg2.0	3,135,165,531	233,566	84.60%
wtdbg2.4	3,105,429,266	239,655	85.10%
wtdbg2.6	3,202,933,435	222,469	85.30%

**Table 2:** Assembly statistics of *R. venosa* genome.

Genome Name	Before Hi-C	After Hi-C
Seq Type	Contig	Scaffold
Total Number	17,949	5,242
Total Length (bp)	2,293,821,241	2,300,182,741
N50 (bp)	434,100	64,632,560
N90 (bp)	434,100	43,368,723
Max Length (bp)	5,188,507	129,259,876
Min Length (bp)	2,089	2,089
Gap Length (bp)	0	6,361,500
GC Content (%)	42.38	42.38

**Table 3:** Statistical result of BUSCO evaluation results of genome assembly.

	Gene number	Percentage
Complete BUSCOs (C)	886	90.6%
Complete and single-copy BUSCOs (S)	832	85.1%
Complete and duplicated BUSCOs (D)	54	5.5%
Fragmented BUSCOs (F)	18	1.8%
Missing BUSCOs (M)	74	7.6%
Total BUSCO groups searched	978	100%

### Chromosomal-level genome scaffolding with Hi-C data

**Fig. 2:**
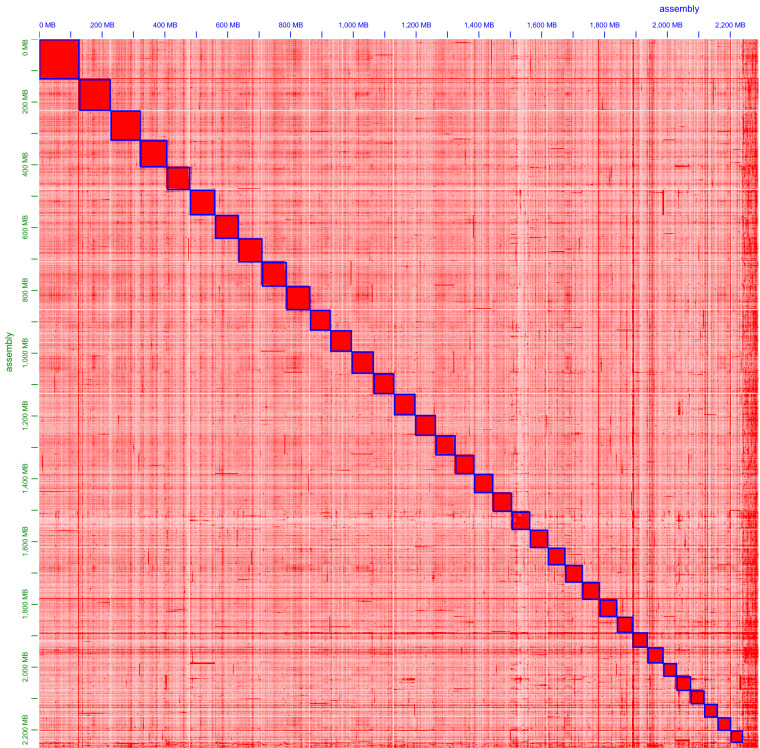
Hi-C assembly of chromosome interactive heat map. Abscissa and ordinate represent order of each bin on corresponding chromosome group. Color block illuminates intensity of interaction from white (low) to red (high).

In total, 4991.96 million read pairs raw data were obtained from the Hi-C sequencing. We conducted quality control, sorting, and duplication removal using HiC-Pro v. 2.8.0^29^. Using the Burrows-Wheeler Aligner (v. 0.7.10-r789)^30^, 63.86% of the clean data were aligned to the draft genome assembly. Here, after using Juicer v1.5^31,32^ and 3D-DNA v170123^33^ to infer order and orientation, 97.88% of the contigs could be placed into 35 scaffolds (chromosomes), with their lengths ranging from 35.91 Mb to 129.26 Mb (Fig. 1, Table 4). After Hi-C scaffolding, the final *Rapana* genome assembly had a size of 2,251.40 Mb and a scaffold N50 of 64.63 Mb (Table 2). A chromatin contact matrix was manually curated in Juicebox v1.5^34^ and the 35 scaffolds are clearly distinguishable in the heatmap in Fig. 2; the interaction signal around the diagonal is strongly apparent.

**Table 4:** Statistics of *R. venosa* genome sequence length (chromosome level).

Chromosome ID	Length (bp)	Percentage
1	129,259,876	5.62
2	102,937,426	4.48
3	93,021,499	4.04
4	86,596,267	3.76
5	84,105,318	3.66
6	78,962,513	3.43
7	75,945,163	3.30
8	75,018,702	3.26
9	74,270,578	3.23
10	71,298,014	3.10
11	68,941,643	3.00
12	68,591,446	2.98
13	67,222,234	2.92
14	66,967,177	2.91
15	64,632,560	2.81
16	63,771,600	2.77
17	63,638,393	2.77
18	61,408,853	2.67
19	60,192,790	2.62
20	59,395,429	2.58
21	59,572,819	2.59
22	58,189,201	2.53
23	56,867,690	2.47
24	56,540,346	2.46
25	55,602,779	2.42
26	55,382,934	2.41
27	52,294,145	2.27
28	49,418,871	2.15
29	45,417,075	1.97
30	44,063,510	1.92
31	43,368,723	1.89
32	43,032,484	1.87
33	41,114,260	1.79
34	38,441,582	1.67
35	35,913,348	1.56
Total	2,251,397,248	97.88
Unplaced	48,785,493	2.12

### Repeat sequences and genome annotation

We used *ab initio* prediction and homology comparison to annotate the repetitive *R. venosa* genomic elements. For the *ab initio* repeat annotation, we used RepeatModeler v. 1.0.9^35^, LTR_FINDER v. 1.0.7^36^, and RepeatScout v. 1.0.7^37^ to build a *de novo* repetitive element database. We used RepeatMasker v. 4.0.7^38^ to annotate the repeat elements in the database. We used RepeatMasker v. 4.0.7 and RepeatProteinMask v 4.0.7 to identify the known repeat element types via searching the Repbase v. 20181026^39^. In addition, Tandem Repeats Finder (TRF v. 4.09)^40^ was used to annotate tandem repeats, identifying 1327.65 Mb of repetitive sequences, representing 57.72% of the assembled genome. This proportion is substantially higher than in closely related species, such as *Lottia gigantea* (10.39%)^41^, *Aplysia californica* (21.80%)^42^, *P. canaliculata* (11.27%)^25^, and *C. bullatus* (38.56%)^4^. Among the repeat sequences, long interspersed nuclear elements were dominant (911.70 Mb, 39.636% of the assembled genome), and short interspersed nuclear elements were the rarest (6.09 Mb, 0.27%) (Table 5).

**Table 5:** Classification of repeat elements in the *R. venosa* genome.

Type	Repbase TEs		Protein TEs		Denovo TEs		Combined TEs	
	Length(bp)	% of genome	Length(bp)	% of genome	Length(bp)	% of genome	Length(bp)	% of genome
DNA	434,449,483	18.888	785,583	0.034	402,828,966	17.513	674,041,712	29.304
LINE	177,491,372	7.716	137,364,314	5.972	822,399,881	35.754	911,702,236	39.636
SINE	1,911,991	0.083	0	0	4,221,194	0.184	6,094,063	0.265
LTR	101,206,291	4.4	2,408,799	0.105	552,520,316	24.021	606,176,386	26.353
Other	47,955	0.002	0	0	0	0	47,955	0.002
Unknown	0	0	0	0	3,681,471	0.16	3,681,471	0.16
Total	566,012,533	24.607	140,548,746	6.11	1,296,949,871	56.385	1,327,648,628	57.719

Candidate non-coding RNAs were annotated as follows. Ribosomal and transfer RNAs were predicted through BLASTN v. 2.2.28^43^ and tRNAscan-SE v. 1.4^44^ (www.lowelab.ucsc.edu/tRNAscan-SE/), respectively. We thus annotated 165 rRNA and 3,241 tRNA genes (e-value: 1e^–10^). We searched against the Rfam database using Infernal v. 1.1.2^45^ (http://infernal.janelia.org/) and identified 76 micro and 103 small nuclear RNAs.

We applied *de novo*, homolog-based, and transcriptomic strategies to annotate the protein coding genes in the *R. venosa* genome. For the *de novo* prediction, Augustus v. 3.2.3^46^, pre-trained using the transcripts assembled from the RNA-seq of *R*. venosa, was employed to predict the coding regions on the repeat-masked assembly. The optimal parameters were obtained after the model training. For the homology-based prediction, we first downloaded the protein sequences of closely related molluscan species, including *L. gigantea*, *C. consors*, *P. canaliculata*, *A. californica*, *A. immaculata*, *Elysia chlorotica*, *B. glabrata*, *C. gigas*, *Octopus vulgaris*, and *Haliotis rubra* from the NCBI database. These protein sequences were aligned against the genome assembly using BLAT v. 35^47^ with an e-value threshold of 1e^−5^. Then, we used GeneWise v. 2.4.1^48^ to align the matching proteins to the homologous genomic sequences to accurately splice the alignments. For the transcriptomic prediction, Hisat v. 2.0.4^49^ and Stringtie v. 1.2.3^50^ were used for assembly based on the reference transcripts, and TransDecoder v. 5.5.0 (https://i5k.nal.usda.gov/Tigriopus_californicus) was used for gene prediction. Finally, all results were merged to form a consensus gene set using GLEAN^51^, and 29,649 protein-coding genes were predicted. To functionally annotate the protein-coding genes, we searched public biological functional databases (SwissProt, InterPro, KEGG, and TrEMBL) for their sequences using BLASTX v. 2.2.28^43^ and BLASTN v. 2.2.28^43^ with an e-value threshold of 1e^−5^; 22,894 genes (77.22%) were annotated in at least one public database.

## Data Records

The raw Illumina, PacBio, and Hi-C sequencing data are deposited in the NCBI SRA database under the accession numbers SRR22889214^52^, SRR23517974^53^, SRR23501451^54^, SRR23501452^55^, SRR23501453^56^, and SRR23501454^57^, respectively. The genome assembly has been deposited in the NCBI SRA database under the accession number JAQIHA000000000^58^. The genome annotations are available from the Figshare repository^59^.

## Technical Validation

### Evaluating genome assembly and annotation completeness

**Fig. 3:**
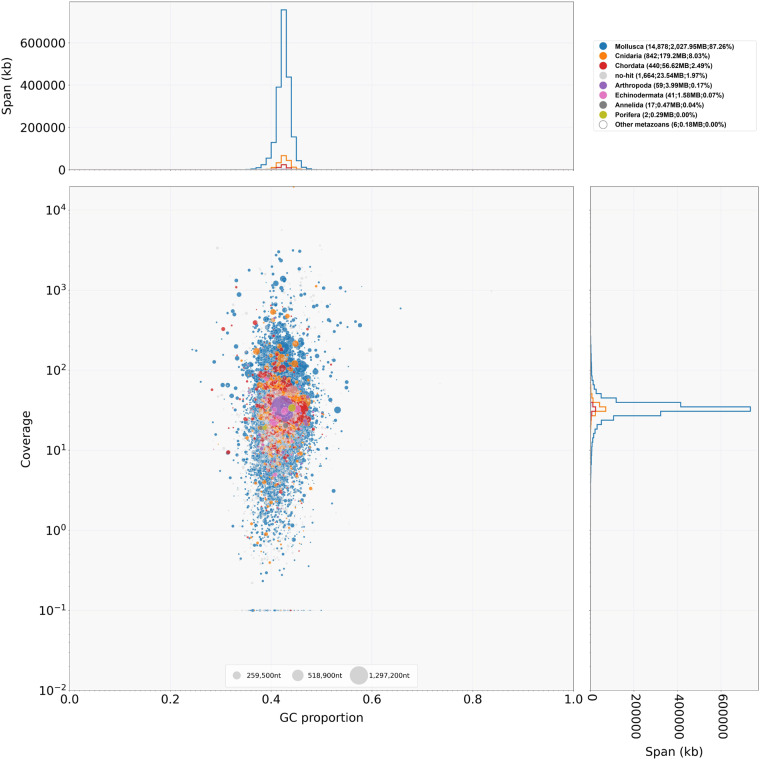
Taxon-annotated GC-coverage plot (BlobPlot) of the contigs used for *R. venosa* genome assembly. Each circle represents a contig sequence, plotted relative to its base coverage and GC proportion. Circle diameter is proportional the size of the contig it represents. Circles are colored according to their assigned taxon at the phylum level (see legend). Histograms show the distribution of the total assembly length along each axis.

The assembled *R. venosa* genome size is 2.30 Gb with a scaffold N50 of 64.63 Mb (Fig. 1), close to the estimated size in previous studies^22^. Using blobtools v. 1.1.1^60^, we created a blobplot to evaluate possible contamination of the contigs used for genome assembly (Fig. 3). As a result, we determined that 87.26% of the contigs had BLAST hits to mollusca. The remaining 12.74% of the contigs were categorized as follows: 8.03% as cnidaria, 2.49% as chordata, 0.17% as arthropoda, 0.07% as echinodermata, 0.04% as annelida, and 1.97% did not match any taxonomic group. These results suggest that the contigs used for *R. venosa* genome assembly were not contaminated with microorganisms. For the quality assessment of the genome assembly, an 90.6% completeness of BUSCO was obtained. The protein-coding sequence possessed an 89.1% completeness of BUSCO. These results suggest a high-quality *R. venosa* genome assembly considering its high heterozygosity and repeat content. The Illumina short reads were mapped to the assembled genome using BWA v. 0.7.10 to evaluate the completeness of the genome assembly^30^. As shown in the Tables 6, 99.30% of the reads could be mapped, covering 78.51% of the assembled genome (Table 6). The Hi-C heatmap shows a well-organized interaction pattern within the chromosomal region (Fig. 2), and assembly resulted in 35 chromosome-level scaffolds, in line with previously published karyotyping^48^. Taken together, these confidently confirm the accuracy of the chromosome scaffolding.

**Table 6:** Statistical results of short read alignment.

	Result
Read mapping rate (%)	99.30
Genome average sequencing depth (×)	27.25
Coverage of genome (%)	78.51
Coverage of genome > 4 × (%)	68.79
Coverage of genome > 10 × (%)	59.70
Coverage of genome > 20 × (%)	48.09

### Collinearity analysis and phylogenetic analysis

**Fig. 4:**
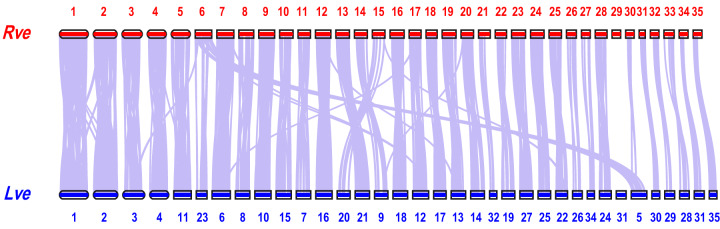
Genomic synteny between *R. venosa* and *L. ventricosus*.

Collinearity analysis of chromosomes between *R. venosa* and another Caenogastropoda species *Lautoconus ventricosus*^61^ was conducted with LASTZ v. 1.02.00^62^. As shown in Fig. 4, almost 35 chromosome-level scaffolds of *R. venosa* displayed high homology with the corresponding chromosomes of *L. ventricosus*, which is suggestive of high quality sequencing and assembly and also make phylogenetic analysis more reliable. For phylogenetic analysis, we conducted pairwise sequence comparisons to predict orthologous genes. First, BLASTP v. 2.2.28 with an e-value cutoff of 1e^–7^ was used to compare the protein sequences of all species. Then, TreeFam v. 9^63^ was applied to cluster all genes. The species used in the gene family clustering analysis were *R. venosa*, *H. rubra*, *L. gigantea*, *C. consors*, *P. canaliculata*, *A. californica*, *A. immaculata*, *E. chlorotica*, *B. glabrata*, *C. gigas*, and *O. vulgaris*.

**Fig. 5:**
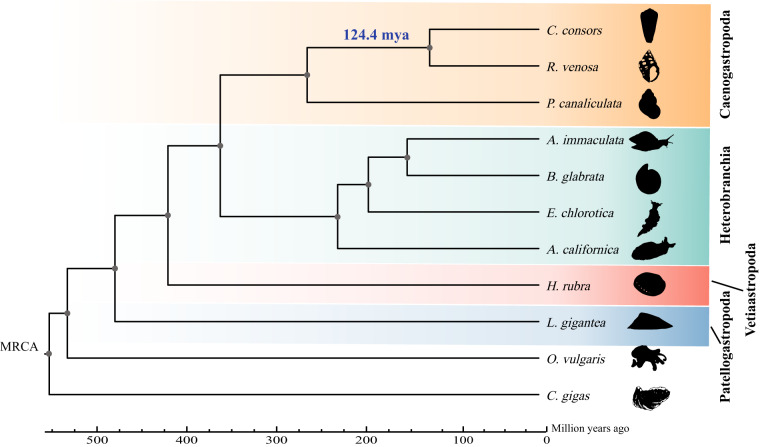
Phylogenetic analysis of *R. venosa* and 10 other species.

Phylogenetic trees were constructed based on single-copy orthologous gene families. Based on the alignment results of the orthologous protein sequences in MUSCLE v. 5.1^64^, the corresponding coding regions of these protein sequences were selected. We extracted the fourfold degenerate synonymous sites of each alignment and concatenated them to form an individual supergene for each species. We used the supergene alignments to perform a maximum likelihood tree using PhyML v. 2.4.4^65^, Mrbayes v. 3.2.6, and RAxML v. 8.2.12^66^, respectively. Finally, the tree was visualized using Figtree (Fig. 4a). The phylogenetic tree shows that *R. venosa* and *C. consors* cluster into one clade, and the positions of the other clades are consistent with previously findings^26^. MCMCtree^67^ in PAML v. 4.4b^68^, with a correlated molecular clock and HKY85 substitution model, was selected to estimate the divergence times between species. Five calibration nodes were used: *C. gigas* and *O. vulgaris* 532–582 mya, *H. rubra* and *P. canaliculata* 401–507 mya, *L. gigantea* and *A. californica* 401–507 mya, *R. venosa* and *P. canaliculata* 155–508 mya, and *E. chlorotica* and *C. consors* 334–489 mya. The divergence times of the calibrated nodes were retrieved from the TimeTree website (http://www.timetree.org/). As shown in the phylogenetic tree, the estimated split time between *R. venosa* and *C. consors* was approximately 124.4 mya (Fig. 5).

## Data Availability

No custom code was used in this study. The data analyses used standard bioinformatic tools specified in the methods.
